# Involvement of the Toll-Like Receptor/Nitric Oxide Signaling Pathway in the Pathogenesis of Cervical Cancer Caused by High-Risk Human Papillomavirus Infection

**DOI:** 10.1155/2017/7830262

**Published:** 2017-05-24

**Authors:** Jie Li, Heping Rao, Chang'e Jin, Jinrong Liu

**Affiliations:** ^1^Department of Infectious Disease, The Second Affiliated Hospital and Yuying Children's Hospital of Wenzhou Medical University, Wenzhou 325027, China; ^2^Department of Nursing, School of Medicine, Quzhou College of Technology, Quzhou 324000, China; ^3^Intensive Care Unit, Laigang Hospital Affiliated to Taishan Medical University, Laiwu 272009, China; ^4^Department of Pediatrics, The Second Affiliated Hospital and Yuying Children's Hospital of Wenzhou Medical University, Wenzhou 325027, China

## Abstract

Human papillomavirus (HPV) can activate Toll-like receptor (TLR)/nitric oxide (NO) signaling pathways; however, whether the TLR/NO pathway is involved in cervical cancer caused by high-risk HPV (HR-HPV) remains unclear. In this study, 43 HR-HPV-positive patients with cervical cancer (CC group), 39 HR-HPV-positive patients with a healthy cervix (HR-HPV group), and 33 HR-HPV-negative controls were recruited. NO concentration in cervical canal and expression of inducible NO synthase (iNOS) in cervical tissues were detected. Expressions of key TLR/NO pathway genes (TLR3/4/7/8, NF-*κ*B p65, and iNOS) in cervical epithelial cells were detected by quantitative reverse transcription PCR. Expressions of TLR4, NF-*κ*B p65, and iNOS in CaSki, HeLa, and C33a cells were determined by Western blot. NO concentration in cervical canal of CC group was significantly higher than in other groups (*P* < 0.05). Positive rates of iNOS in cervical tissues were 72.1%, 28.2%, and 3.1% in the CC group, HR-HPV group, and controls, respectively (*P* < 0.05). Levels of TLR3, TLR4, TLR7, TLR8, NF-*κ*B p65, and iNOS in cervical epithelial cells were higher in CC group than in other groups (*P* < 0.05). Both mRNA and protein levels of TLR4, NF-*κ*B p65, and iNOS were higher in HPV-positive HeLa and CaSki cells than in HPV-negative C33a cells (*P* < 0.05). Together, these results suggest that TLR/NO signaling pathway may be involved in pathogenesis of cervical cancer caused by HR-HPV.

## 1. Introduction

Cervical cancer is one of the most common gynecologic malignant tumors. The incidence of cervical cancer is the third highest among all female malignant tumors worldwide, and its mortality rate is high [1, 2]. High-risk human papillomavirus (HR-HPV) infection is a major risk factor of cervical cancer; persistent infection with HR-HPV can cause cervical cancer and precancerous lesions [3]. In particular, HPV16 and HPV18 are the two types of HR-HPV which are closely related with cervical cancer [3, 4]. However, the specific mechanism of cell malignant transformation caused by HR-HPV infection is still unclear.

HPV can be eliminated by autoimmunity in 90% of HPV-infected patients. Among the remaining chronically infected patients, only 1% will finally develop cervical cancer [5, 6]. As HR-HPV mainly infects the cervical epithelial cells, the antiviral effect of immune cells in the peripheral blood is very limited [7]. Therefore, exploring the role of local cervical immune regulation factors in HR-HPV infection is of great significance and clinical value.

Toll-like receptors (TLRs) are the most distinctive pathogenic pattern recognition receptors involved in the defense against infection in humans [8]. In addition, TLRs are widely expressed in a variety of human malignant tumor tissues [8]. Among human TLR family members, TLR2, 3, 4, 7, and 9 have clear associations with tumors, while TLR3, 4, 7, 8, and 9 are related to virus recognition [8, 9]. HPV oncogene proteins or HPV virus-like particles can regulate host immunity via TLR pathways through a variety of unclear mechanisms [10, 11]. Therefore, TLRs play a crucial role in cervical HR-HPV infection and the process of carcinogenesis.

In recent years, the tumor microenvironment has become a hot topic in oncology research. Nitric oxide (NO) is one of the key components in the formation of the tumor microenvironment caused by chronic infection [12]. Inducible nitric oxide synthase (iNOS) is the main enzyme for the synthesis of NO. This enzyme is highly expressed in many kinds of malignant tumors, resulting in the catalytic synthesis of a large amount of NO [12, 13]. In turn, NO promotes tumor angiogenesis, as well as tumor cell invasion and metastasis [12, 13]. Indeed, high iNOS expression in cervical cancer is positively correlated with tumor malignancy and lymph node metastasis [14].

The binding of TLR with its specific ligand has been shown to trigger signal transduction cascades, activate nuclear factor kappa B (NF-*κ*B), regulate iNOS, and mediate NO secretion through the myeloid differentiation factor 88 (MYD88) or TIR-domain-containing adapter-inducing interferon-*β* (TRIF) pathway [15, 16]. However, whether the TLR/NO signaling pathway is involved in the pathogenesis of cervical cancer caused by HR-HPV infection remains unclear. This study aims to investigate the role of the TLR/NO signaling pathway in HR-HPV-positive cervical cancer.

## 2. Materials and Methods

### 2.1. Subjects

Forty-three patients with HR-HPV-positive cervical squamous carcinoma were recruited as the cervical cancer group from Department of Infectious Disease, the Second Affiliated Hospital and Yuying Children's Hospital of Wenzhou Medical University, from January to October 2015. The age range of the patients was 31–59 years, with a median age of 41.3 years, including 19 cases < 45 years and 24 cases ≥ 45 years. The tumor size was <4 cm in 25 cases and was ≥4 cm in 18 cases. The clinical stage was determined according to the 2009 International Federation of Gynecology and Obstetrics (FIGO) [17]. Meanwhile, 39 patients with a HR-HPV-positive healthy cervix (diagnosed by pathological examination) and 33 HR-HPV-negative healthy subjects were recruited as the HR-HPV group and the control group, respectively. The age ranges were 24–57 years (median age of 38.6 years) and 20–61 years (median age of 36.9 years) in the HR-HPV group and the control group, respectively.

E6 and E7 mRNA kits (Hybribio Limited, Guangdong, China) were used for the HR-HPV detection, which can detect 14 kinds of HR-HPV (including HPV16, 18, 31, 33, 35, 39, 45, 51, 52, 56, 58, 59, 66, and 68), according to manufacturer's instructions. For detection of the NO concentration in the cervical canal, those patients with local cervical bleeding were excluded and specimens of cervix secretion with cleanliness ≤ I degree were selected. This research was approved by the ethics committee of the Second Affiliated Hospital and Yuying Children's Hospital of Wenzhou Medical University. Written informed consent was obtained from all subjects for participation in the study.

### 2.2. Cell Lines and Reagents

CaSki (HPV16+), HeLa (HPV18+), and C33A (HPV−) cell lines were purchased from the Chinese Academy of Sciences Typical Culture Preservation Committee Cell Bank (Shanghai, China). The NO detection kit was from Beyotime Biotechnology (Shanghai, China). The streptavidin-peroxidase (SP) immunohistochemical staining kit was from Mingrui (Shanghai, China). The mRNA isolation kit, TRIzol kit, fetal bovine serum (FBS), and DMEM were from Thermo Fisher (Waltham, MA). Rabbit anti-human TLR4 polyclonal antibody, mouse anti-human NF-*κ*B p65 monoclonal antibody, rabbit anti-human iNOS polyclonal antibody, and mouse anti-human GAPDH monoclonal antibody were from Cell Signaling Technology (Danvers, MA).

### 2.3. Detection of the NO Concentration in the Cervical Canal

Patients lay in the lithotomy position. The cervix was exposed with a speculum, and the cervical secretion was wiped. A cotton swab was placed into the cervical canal about 1 cm for 20 s and then washed in the glass tube containing 1.5 mL double-distilled water for 2 min. The NO concentration was indirectly detected using the Griess method, as described in the instructions for the NO detection kit. The absorbance at 540 nm was determined with an iMark/xMark microplate reader (Bio-Rad, Hercules, USA). A standard curve was drawn (according to the standard concentration and corresponding absorbance values), and then the concentration of the sample was calculated.

### 2.4. Immunostaining

Expression of iNOS protein was detected by the SP immunostaining kit, according to manufacturer's instructions. Briefly, the histologic material fixed in 10% formalin and embedded in paraffin was cut into 3 *μ*m slices. After deparaffinization, each specimen was incubated for 5 min with 3% hydrogen peroxide, followed by overnight incubation with anti-iNOS polyclonal antibody (1 : 50) at 4°C. The specimens were incubated with biotinylated secondary antibodies for 10 min, followed by treatment with horseradish peroxidase-labeled streptavidin for 10 min. A section was considered negative or positive according to the absence or presence of positive yellow or brown staining. The staining based on positive cells was scored as follows: no positive cells (0), 1%–10% (1), 11%–50% (2), 51%–80% (3), and >81% (4). The staining based on color was scored as follows: no positive cells (0), light yellow (1), brownish-yellow (2), and brown (3). The final score was the multiplication of the above two scores. The positive or negative expression was determined by the final score of 0–2 (−) and >2 (+). The positive rate was then calculated.

### 2.5. Quantitative Reverse Transcription- (RT-) PCR

The expression of key genes in the TLR/NO signaling pathway in CaSki, HeLa, and C33a cells was detected by quantitative RT-PCR. Total RNA was extracted using TRIzol reagent from cells. cDNA was then synthesized from a total of 100 ng extracted RNA, followed by PCR using a quantitative PCR kit according to the manufacturer's instructions. All primers used are listed in Table 1. The reaction mix included 10 *μ*L SYBR Green Master Mix, 0.2 *μ*L each of forward and reverse primers, and 2 *μ*L cDNA and was taken to a final volume of 20 *μ*L with water. Quantitative PCR was performed for 40 cycles of denaturation (95°C for 45 s), annealing (62°C for 30 s), and extension (72°C for 30 s); dsDNA was measured at 86°C after each cycle. All the data of the triplicate experiments were expressed relative to glyceraldehyde-3-phosphate dehydrogenase (GAPDH) expression. Fold change in the relative gene expression to control was determined by the standard 2^−ΔΔ*C*_*t*_^ method.

### 2.6. Western Blot Analysis

Approximately 1 × 10^6^ cells were lysed in 200 *μ*L cell lysis buffer (Thermo Fisher Scientific, Waltham, USA) for 30 min at 4°C and then clarified by centrifugation at 8000*g* for 10 min. Protein concentration of each sample was determined using a protein assay kit (Pierce, Rockford, ILUSA), with bovine serum albumin as the standard. Western blotting was performed as previously described [18, 19]. Briefly, 10 *μ*g of cell protein underwent SDS-PAGE, followed by transfer to a polyvinylidene difluoride membrane (Millipore, Shanghai, China). After blocking with 5% nonfat milk powder in 50 mM Tris-HCl (pH 7.6), 150 mM NaCl, and 0.1% Tween 20, 1-2 *μ*g/mL of primary antibody was added and incubated with the blots overnight at 4°C. After washing and incubation with horseradish peroxidase-labeled secondary antibodies for 2 h, the membrane was imaged using a VersaDoc MP 5000 system (Bio-Rad). GAPDH was used as an internal reference using an anti-human GAPDH polyclonal antibody (Cell Signaling Technology).

### 2.7. Statistical Analysis

Every experiment was repeated three times with duplicates, and data are reported as the mean ± SD. Statistical significance of the results was calculated using SPSS v.20 software (SPSS Inc., Chicago, USA). Student's *t*-test was used to compare values between the two groups; one-way analysis of variance was used when comparing more than three groups. Statistical significance was set at a level of *P* < 0.05.

## 3. Results

### 3.1. The Clinical Characters of the Cervical Cancer Group

According to the FIGO staging criteria, 17 cases (39.5%) of the 43 cervical cancer patients were phase I, 8 cases (18.6%) were phase II, and 18 (41.9%) cases were phase III. Sixteen cases (37.2%) had lymph node metastasis, 21 cases (48.8%) had lymphatic vascular invasion, and 6 cases (14.0%) had both. The detailed information was shown in Table 2.

### 3.2. Comparison of NO Concentration in the Cervical Canals

**Figure 1:**
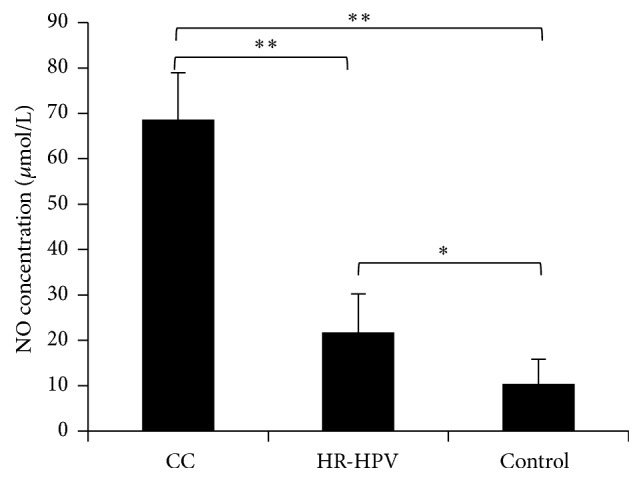
NO concentration in the cervical canal of HR-HPV-positive patients and healthy controls. CC: HR-HPV-positive cervical cancer patients; HR-HPV: HR-HPV-positive patients; Control: healthy controls. ^*∗*^*P* < 0.05, ^*∗∗*^*P* < 0.01.

We first tested the NO concentration in the cervical canals of patients in the cervical cancer group, the HR-HPV group, and the control group. The NO concentration in the cervical canal was 68.63 ± 10.34 *μ*mol/L in the cervical cancer group, significantly higher than that in the HR-HPV group (21.71 ± 8.49 *μ*mol/L; *P* < 0.01) and the control group (10.38 ± 5.42 *μ*mol/L; *P* < 0.01). In addition, there was significant difference between the HR-HPV group and the control group (*P* < 0.05; Figure 1).

### 3.3. Comparison of iNOS Protein Expression in the Cervical Tissue

**Figure 2:**
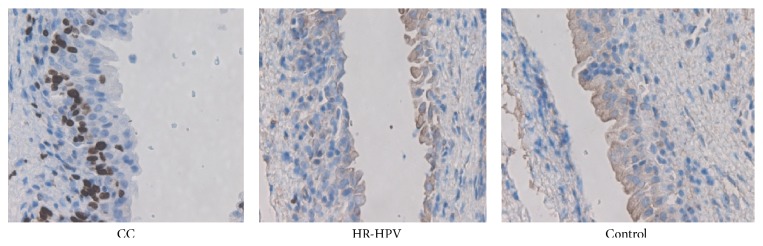
iNOS protein expression in the cervical tissue of HR-HPV-positive patients and healthy controls. iNOS protein expression was detected with the immunohistochemical method. CC: HR-HPV-positive cervical cancer patients; HR-HPV: HR-HPV-positive patients; Control: healthy controls.

Immunohistochemical results showed that no tan or brown staining was observed in the cervical tissue of most healthy controls and that iNOS protein expression was negative (Figure 2). A small amount of tan or brown staining was observed in the cervical tissue of the HR-HPV group. Strong brown staining was observed in the nucleus and cytoplasm of cervical tissue in most HR-HPV-positive cervical cancer patients, and iNOS protein expression was positive. The iNOS protein positive expression rates were 72.1% (31/43), 28.2% (11/39), and 3.1% (2/33) in the cervical cancer group, HR-HPV group, and control group, respectively (*P* < 0.05).

### 3.4. Comparison of mRNA Expression of Key TLR/NO Signaling Pathway Genes in the Cervical Tissue Epithelial Cells

**Figure 3:**
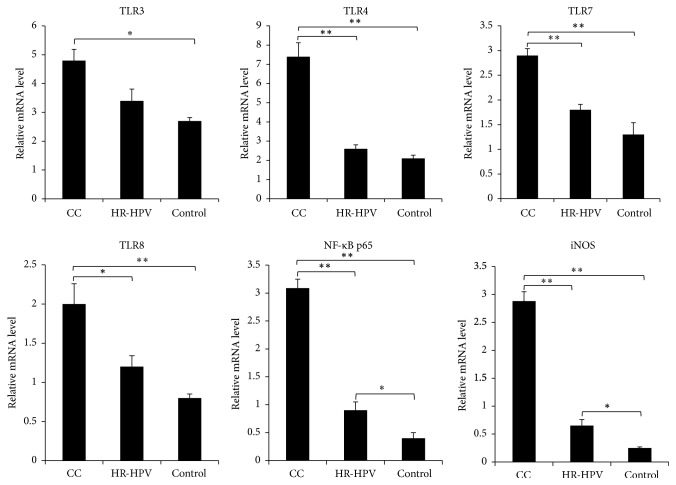
Expression of key TLR/NO signaling pathway genes in cervical epithelial cells of HR-HPV-positive patients and healthy controls. mRNA expression levels were detected with quantitative RT-PCR. CC: HR-HPV-positive cervical cancer patients; HR-HPV: HR-HPV-positive patients; Control: healthy controls. ^*∗*^*P* < 0.05; ^*∗∗*^*P* < 0.01.

We tested the TLR3, TLR4, TLR7, TLR8, NF-*κ*B p65, and iNOS mRNA expression levels in the cervical tissue epithelial cells of the three groups and found that all mRNA levels were higher in the cervical cancer group than in the HR-HPV group and control group (*P* < 0.05; Figure 3). Of the four kinds of TLRs, the most significant difference was found in TLR4 expression, which was upregulated by 3.1–4.5 times in the cervical cancer group compared with the control group (Figure 3). There was no obvious difference in the TLR expression levels between the HR-HPV group and the healthy control group; however, the NF-*κ*B p65 and iNOS mRNA expression levels were significantly upregulated in the HR-HPV group (*P* < 0.05; Figure 3).

### 3.5. Comparison of mRNA and Protein Expression of Key TLR4/NO Signaling Pathway Components in CaSki, HeLa, and C33a Cells

**Figure 4:**
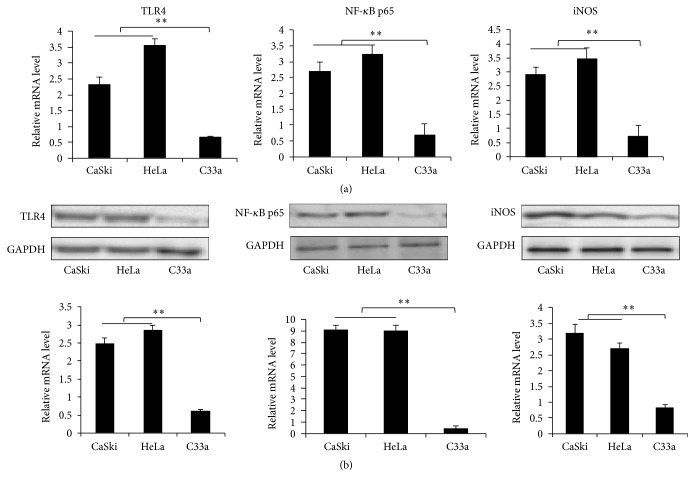
Expression of key TLR4/NO signaling pathway genes in CaSki, HeLa, and C33a cells. (a) mRNA expression levels were detected with RT-PCR, and (b) protein levels were detected by Western blot. CC: HR-HPV-positive cervical cancer patients; HR-HPV: HR-HPV-positive patients; Control: healthy controls. ^*∗*^*P* < 0.05; ^*∗∗*^*P* < 0.01.

We further verified the activation of the TLR4/NO signaling pathway in cervical cancer cell lines, including CaSki (HPV16+), HeLa (HPV18+), and C33a (HPV−) cells. First, we detected mRNA expression levels with quantitative RT-PCR and found that the TLR4, NF-*κ*B p65, and iNOS mRNA expression levels were higher in HPV-positive HeLa and CaSki cells than in HPV-negative C33a cells (*P* < 0.01; Figure 4(a)). Western blotting results showed that the TLR4, NF-*κ*B p65, and iNOS protein expression levels were also higher in HeLa and CaSki cells than in C33a cells (*P* < 0.01; Figure 4(b)).

## 4. Discussion

In this study, we investigated the role of the TLR/NO signaling pathway in HR-HPV-positive cervical cancer. We found that the expressions of key genes and proteins in the TLR4/NO signaling pathway were upregulated in HR-HPV-positive cervical cancer tissues and cells, suggesting that TLR4/NO signaling pathway activation may participate in the pathogenesis of cervical cancer caused by HR-HPV infection.

The expression of iNOS occurs during inflammation, cancer, and other pathological conditions [20]. Its expression can be induced by NF-*κ*B activation in tumor cells, immune cells, and other cells, thus synthesizing a large amount of NO [15, 16]. Previous studies revealed that the NO concentration in the cervical canal was significantly higher in HR-HPV-infected individuals than in those not infected or with low-risk HPV infection [21]. This suggests that the NO concentration in the cervical canal is closely related to HR-HPV infection [21]. Furthermore, when HPV-positive cells are exposed to NO, the HPV early transcription level is upregulated by 2–4 times, E6 and E7 protein expression levels are significantly upregulated, and the frequency of cell mutation is also increased [22]. Together, these findings suggest that NO may be involved in the carcinogenesis process caused by HPV infection. Indeed, our results confirm that iNOS is highly expressed in the cervical cancer tissue of patients with HR-HPV infection and that the NO concentration in the cervical canal is significantly increased. Therefore, high NO concentrations in the cervical canal appear to be triggered by high iNOS expression that is induced by HR-HPV.

Chronic inflammation is closely related to the pathogenesis of tumors [23]. Pathogens in the vagina can promote TLR and integrin protein expression in the cervix, thereby inducing the occurrence of cervical cancer, confirming that inflammation is an important trigger factor for cervical cancer [24]. In recent years, the role of TLRs in tumorigenesis has attracted more and more attention. In our study, the expression levels of TLR3, TLR4, TLR7, TLR8, NF-*κ*B p65, and iNOS mRNA in the cervical squamous epithelial cells were significantly higher in the cervical cancer group than in the HR-HPV patients and healthy controls. In addition, although there was no obvious difference in TLR expression levels between the HR-HPV and healthy control groups, the NF-*κ*B p65 and iNOS mRNA expression levels were increased significantly in HR-HPV patients. This suggests that HR-HPV may mediate the activation of NF-*κ*B p65 and iNOS pathways through receptors other than TLRs.

The TLR3 pathway can be activated by many viruses, such as influenza A (H1N1), simian immunodeficiency, and measles [25–28]. In this study, the level of TLR3 mRNA expression was obviously upregulated in the cervical squamous epithelial cells in the cervical cancer group. This indicates that the immunomodulatory mechanism of the TLR3 pathway in HR-HPV-positive cervical cancer may be similar to the above viruses. With regard to TLR7 and TLR8, previous studies have found that the application of TLR7 and TLR8 agonists has antitumor activity [29]. However, the specific roles of TLR7 and TLR8 in cervical cancer are yet to be elucidated. In our study, upregulation of TLR4 in cervical cancer was the most significant among the TLRs examined (including TLR3, TLR4, TLR7, and TLR8). By mediating inflammation, TLR4 can regulate the release of a large number of cytokines and proteolytic enzymes. In addition, TL4 can participate in the regulation of the tumor microenvironment and promote the transformation of tumor cells. Indeed, a previous study found that TLR4 was involved in the formation of intestinal inflammatory-related tumor microenvironment [30]. In addition, TLR4 activation can promote tumor growth and resistance to chemotherapy and mediate the immune escape of epithelial ovarian cancer cells [31]. Hasimu et al. [32] found that TLR4 expression was upregulated in cervical cancer in Uygur women of China and positively correlated with HPV16 infection. But a few studies also showed that TLR4 expression was downregulated in cervical cancer, suggesting that HPV can inhibit TLR4 expression [33].

In the current study, we found that the expression levels of key molecules in the TLR4/NO signaling pathway (including TLR4, NF-*κ*B p65, and iNOS) were significantly upregulated in HR-HPV-positive cervical cancer tissues and were also significantly higher in HR-HPV-positive cervical cancer HeLa and CaSki cell lines than in the HPV-negative cervical cancer C33a cell line. Together, these results indicate that TLR4/NO signaling pathway activation in cervical cancer is associated with HR-HPV infection and may be involved in the regulation of the local cervical immune microenvironment and the pathogenesis of cervical cancer.

Our results should be interpreted with the following limitations: first, patients with cervical intraepithelial neoplasia (CIN), the precursor of cervical squamous carcinoma, were not included, and more fully designed studies including patients with CIN should be carried out in future; second, this study is a cross-sectional research, and the cause and consequences relation between TLR/NO signaling pathway and cervical cancer should be validated by further studies. For example, the expression of NO and iNO and activtion of TLR/NO signaling pathway should be investigated after elimination of HPV (spontaneous disappearance or cervical conization).

In conclusion, the expression levels of key genes in the TLR4/NO signaling pathway are upregulated in HR-HPV-positive cervical cancer tissue. Based on our results, we speculate that TLR4 pathway activation is associated with HR-HPV infection and may participate in the regulation of the local cervical immune microenvironment and the pathogenesis of cervical cancer. This is a novel immunoregulatory mechanism in the local cervical microenvironment and provides new strategies for the development of cervical cancer immunotherapies and for the elimination of HPV. Despite this, the role and mechanism of the TLR4/NO signaling pathway in other types of HPV infection, as well as the role of other receptor pathways in HPV infection and cervical cancer, require further investigation.

## Figures and Tables

**Table 1:** Primer sequences used in the study.

Gene	Upstream primer (5′-3′)	Downstream primer (5′-3′)
TLR3	CTGATGCTCCGAAGGCT	CGTGCTAAGTTGTTATGCT
TLR4	CCGAAAGGTGATTCTTG	AAGATGATACCAGCACGAC
TLR7	AAAACTCTGCCCTGTGA	TGAGGTTCGTGGTGTTC
TLR8	CTAAACCACAACCCCAA	TTGTCTTCAAGCAGTAA
NF-*κ*B p65	CTGGACCGCTTGGGTAA	TGCTGCCTTTTTGTGCT
iNOS	CTCAAGGCACAGGTCTC	GGGCTTTCTCCACATTGTT
GAPDH	CCATGTTCGTCATGGGTGTG	GGTGCTAAGCAGTTGGTGGTG

TLR, Toll-like receptor; NF-*κ*B, nuclear factor-*κ*B; iNOS, inducible nitric oxide synthase.

**Table 2:** The clinical characters of the cervical cancer group.

	Phase I	Phase II	Phase III
LNM	11	2	3
LVI	5	5	11
Both LNM and LVI	1	1	4

*Note*. LNM, lymph node metastasis; LVI, lymphatic vascular invasion.
